# Use climatic space‐for‐time substitutions with care: Not only climate, but also local environment affect performance of the key forest species bilberry along elevation gradient

**DOI:** 10.1002/ece3.10401

**Published:** 2023-08-16

**Authors:** Inger Auestad, Knut Rydgren, Rune Halvorsen, Ingrid Avdem, Rannveig Berge, Ina Bollingberg, Oline Lima

**Affiliations:** ^1^ Department of Environmental Sciences Western Norway University of Applied Sciences Sogndal Norway; ^2^ Geo‐Ecological Research Group, Department of Research and Collections, Natural History Museum University of Oslo Oslo Norway

**Keywords:** boreal forest, climate change, NMDS, species composition, *Vaccinium myrtillus*, variation partitioning

## Abstract

An urgent aim of ecology is to understand how key species relate to climatic and environmental variation, to better predict their prospects under future climate change. The abundant dwarf shrub bilberry (*Vaccinium myrtillus* L.) has caught particular interest due to its uphill expansion into alpine areas. Species' performance under changing climate has been widely studied using the climatic space‐for‐time approach along elevation gradients, but potentially confounding, local environmental variables that vary along elevation gradients have rarely been considered. In this study, performed in 10 sites along an elevation gradient (200–875 m) in W Norway, we recorded species composition and bilberry performance, both vegetative (ramet size and cover) and reproductive (berry and seed production) properties, over one to 4 years. We disentangled effects of local environmental variables and between‐year, climatic variation (precipitation and temperature), and identified shared and unique contributions of these variables by variation partitioning. We found bilberry ramet size, cover and berry production to peak at intermediate elevations, whereas seed production increased upwards. The peaks were less pronounced in extreme (dry or cold) summers than in normal summers. Local environmental variables explained much variation in ramet size and cover, less in berry production, and showed no relation to seed production. Climatic variables explained more of the variation in berry and seed production than in ramet size and cover, with temperature relating to vegetative performance, and precipitation to reproductive performance. Bilberry's clonal growth and effective reproduction probably explain why the species persists in the forest and at the same time invades alpine areas. Our findings raise concerns of the appropriateness of the climatic space‐for‐time approach. We recommend including both climatic and local environmental variables in studies of variation along elevation gradients and conclude that variation partitioning can be a useful supplement to other methods for analysing variation in plant performance.

## Graphical abstract

How key species such as bilberry will respond to future climate change is of great interest, but the widely used climatic space‐for‐time approach may be flawed, since it is rarely combined with investigation of local environmental variation. Our study of bilberry growth and reproduction along an elevation gradient (200–875 m) demonstrated the usefulness of variation partitioning for disentangling the effects of climatic and environmental variation.

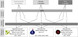

## 1 INTRODUCTION

A major challenge in ecology is to predict responses to the ongoing global climate changes, which are regarded as main threats to ecosystems and key species (IPBES, 2019). In the northern hemisphere, observations of northward and upward migration of common species are interpreted as responses to a warmer climate (Steinbauer et al., 2018). These changes are driven by variations in species' vegetative and/or reproductive performance. While variation in plant biomass and vegetative growth on one hand translates directly into community‐ and ecosystem‐scale variation in species composition (Sundqvist et al., 2013), variation in fruit and seed production, on the other hand, affects the species' dispersal abilities. This may in turn affect their capacity for expansion into previously unoccupied areas (Boscutti et al., 2018).

Alpine and arctic ecosystems are regarded as particularly susceptible to, and disproportionately impacted by, climate change (Parmesan & Yohe, 2003). A noticeable ongoing change is the uphill movement of boreal species in alpine areas, as exemplified by the ericaceous dwarf shrub bilberry (*Vaccinium myrtillis* L.), which, under the ongoing climate warming, migrates into altitudes above its present distribution (Klanderud & Birks, 2003; MacDougall et al., 2021). Bilberry has a wide distribution in Fennoscandian boreal ecosystems (Økland, 1996) and is considered a key species in boreal forests due to its role in trophic networks, considerable biomass production, evergreen twigs and large but variable berry production (Hegland et al., 2010).

The net effect of climate changes on bilberry's performance is not easy to predict since the species responds to both climate means and weather extremes in complex ways (Taulavuori et al., 2013). To better understand and predict bilberry's performance in the boreal as well as alpine zone under the expected climate change, we need to understand how potentially impacting variables affect bilberry performance along elevation gradients.

Elevation is negatively correlated with temperature (Körner, 2007) and, in many parts of the world, also positively correlated with precipitation (Körner, 2007; Pato & Obeso, 2012b). Temperature is a well‐known driver of variation in species' performance in terms of, for example growth and reproduction (Bokhorst et al., 2011; Sundqvist et al., 2013). Observations of responses to short‐term climatic variation (‘weather’) along elevation gradients may therefore be relevant for predicting responses to longer term, broader‐scale climatic changes (Fukami & Wardle, 2005; Walker et al., 2010). This so‐called climatic space‐for‐time substitution approach uses current observations of species' performance along elevation gradients to predict their performance to future climate change (Blois et al., 2013) under the assumption that elevation gradients in space can serve as proxies for climatic gradients in time. The climatic space‐for‐time substitution approach may offer a useful alternative to long‐term monitoring studies, and to manipulative warming experiments (Elmendorf et al., 2015), as the former requires very long data series, and the latter suffers from problems with transferring results from experimental settings to natural systems (Fukami & Wardle, 2005; Walker et al., 2010; Wolkovich et al., 2012).

However, a major limitation of the climatic space‐for‐time substitution approach is that elevation is an indirect gradient (in the sense of Austin, 1980), representing unknown combinations of multiple, direct gradients that generally are not causally related to climatic variation. Examples include many environmental variables that vary on spatially fine scales, such as soil moisture and soil nutrients (Halvorsen et al., 2020; Körner, 2007). Moreover, the climatic space‐for‐time substitution approach has been reported to overestimate the magnitude of climatic impact on species performance (Elmendorf et al., 2015). Elevation should therefore not be used as a proxy for climatic variation unless the effect of potentially confounding factors is controlled for. This is, however, rarely performed. The variation partitioning method (Borcard et al., 1992; Økland, 1999) allows such control by disentangling sources of more or less covarying spatial and temporal variation, for example along elevation gradients, by partitioning of the variation in a data set among different sets of explanatory variables. Variation partitioning has mostly been used for multivariate data (e.g. species composition), but is also applicable to univariate data, such as species' performance variables (Amouzgar et al., 2020; Nielsen et al., 2007; Volis et al., 2011).

Previous studies have demonstrated that bilberry growth and reproductive performance vary along elevation gradients, at local (Pato & Obeso, 2012a) and regional scales (Hertel et al., 2018; Ritchie, 1956; Selås et al., 2015). However, the simultaneous effects of temperature and precipitation on one hand, and of local environmental variables on the other hand, have only occasionally been addressed (Pato & Obeso, 2012a). A fine resolution of the elevation gradient has been adopted in only one study (Pato & Obeso, 2012b), and moreover, no studies have included a broad range of environmental variables.

In this study, we aim to explore, along an elevational gradient, how bilberry performance is influenced by local environmental as well as by climatic variation, the latter varying over time. Moreover, we aim to investigate the use of variation partitioning to disentangle the effects of these sources of variation, uniquely and in concert, on bilberry performance.

Performed along an elevation gradient that ranges from 200 to 875 m a.s.l. in a boreal pine forest in W Norway, this study analyses the relationships between variation in bilberry performance (response variables) and sets of explanatory variables. These include short‐term variation in temperature and precipitation, hereafter termed ‘climatic variation’, and other, locally varying variables, hereafter termed ‘environmental variation’. Bilberry performance variables include ramet size and cover as measures of growth, and berry and seed production as measures of reproduction. Variation partitioning allows sorting of the variation in bilberry performance on climatic, environmental and temporal variation components, both shared and unique.

Based on our findings, we discuss the implications for future studies using the climatic space‐for‐time‐substitution approach along gradients and the usefulness of variation partitioning in this context.

## 2 MATERIALS AND METHODS

### 2.1 Study site

**FIGURE 1:**
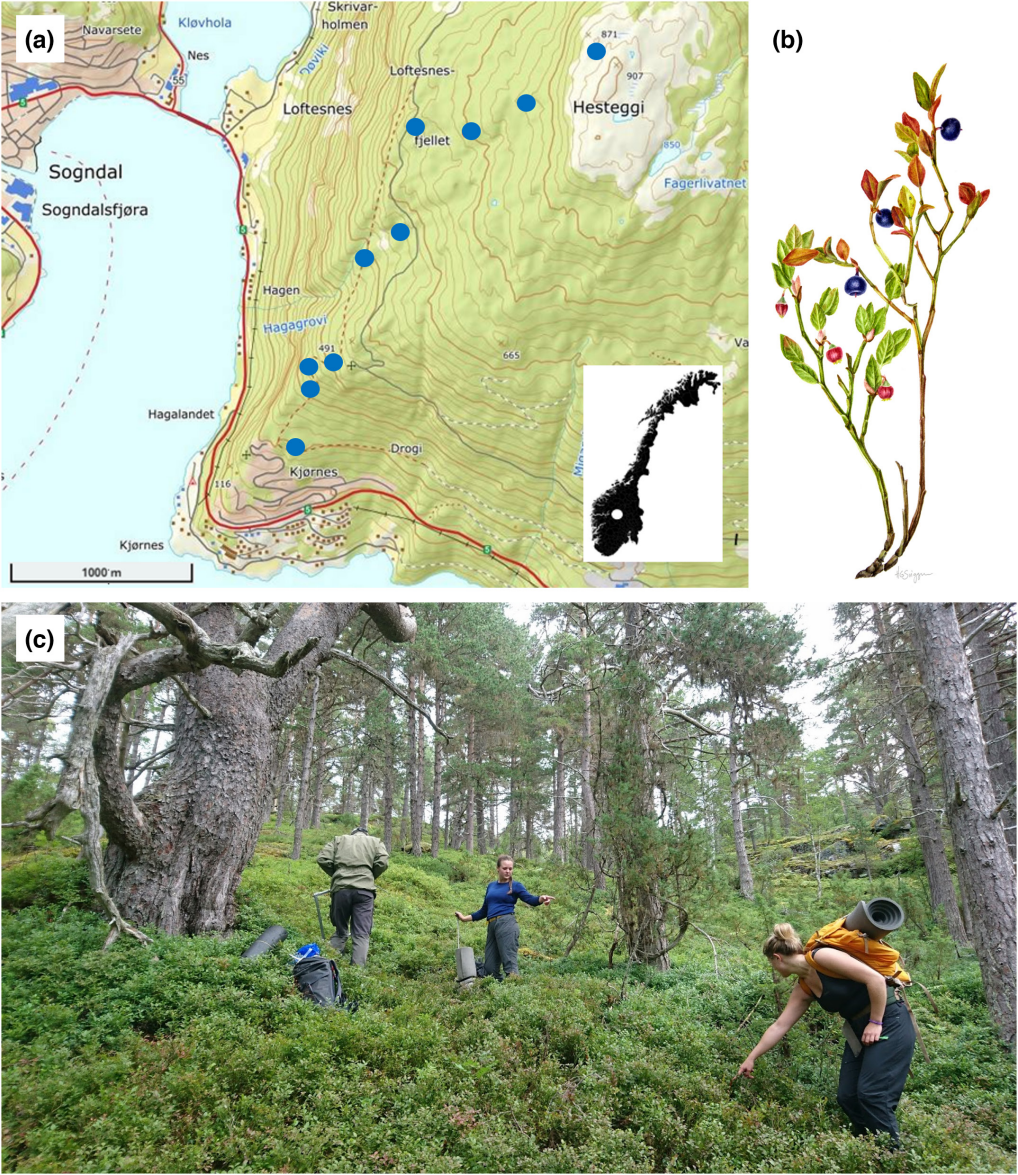
Study area was situated on a SW facing hillside in Sogndal, W Norway, the 10 blocks shown as red dots at elevations between 200 and 875 m, with map of Norway inserted in lower right corner (a). The study species bilberry (b), and the vegetation of the steep, bilberry‐dominated pine forest (c).

We studied bilberry performance in 2017–2020 along an elevation gradient in a boreal, bilberry‐dominated Scots pine (*Pinus sylvestris*) forest in Sogndal, Vestland county, W Norway (61°13′ N, 7°9′ E; Figure 1). The study site extended along a 2.7 km SW‐facing ridge, covering the altitudinal interval 200–875 m a.s.l., from dense lowland forest up to the alpine tree line that occurred just below the mountain top Hesteggi at 907 m a.s.l. The bedrock consisted of Precambrian anorthosite and gneiss, covered by a discontinuous layer of glacial deposits which decreases in thickness with increasing elevation (NGU, 2018). Podzol soils prevail. The site is situated in the weakly oceanic bioclimatic section and spanned the range from the south boreal to the transition between the north boreal and low alpine bioclimatic zones (Bakkestuen et al., 2008). The estimated annual mean temperature at Sogndalsfjøra (2 km W of the study site, at 10 m a.s.l.) was 6.7°C (July mean: 15.0°C, January mean: –1.5°C). Precipitation peaks in autumn, with annual mean precipitation of 1025 mm for the normal period 1961–1990 (met.no, 2020).

The pine forest had a sparse *Juniperus communis* shrub layer, a field layer dominated by dwarf shrubs such as bilberry, *Vaccinium vitis‐idaea*, and *Empetrum nigrum*, and a bottom layer dominated by bryophytes such as *Pleurozium schreberi* and *Hylocomium splendens* (Figure 1c). The area is grazed at low intensities by red deer and sheep.

### 2.2 Study species

Bilberry (*V. myrtillus* L., Figure 1b), is a 10–60‐cm tall, erect dwarf shrub with creeping subterranean rhizomes and evergreen stems (Ritchie, 1956). It is widely distributed in Eurasia (Hultén & Fries, 1986) but has its climatic optimum in N Europe (Coudun & Gégout, 2007). In Fennoscandia, the species is common throughout the boreal forest and dominates low‐alpine heaths. The species mainly propagates clonally and form large genets consisting of ramets connected under the moss mat (Ritchie, 1956). Using ramets as study units, we defined a ramet as ‘all twigs that were connected to one main twig protruding from the moss mat’. The bilberry flowers are pollinated by bumblebees in April–June and produce ripe berries in July–August (Eckerter et al., 2019). It has been referred to as a masting species due to its large interannual variation in berry production (Selås, 2000). Bilberry seeds survive in the soil seed bank for several years, but germination from the seed bank in the boreal forest appears to be limited (Baskin et al., 2000; Rydgren et al., 1998; Welch et al., 2000).

### 2.3 Sampling design and data collection

In 2017, we subjectively placed 10 blocks of 5 × 10 m along the elevation gradient, all facing S–SW with an average slope of 10°–30°. Each block spanned the variation from open to shaded sites. In each block, we randomly placed and permanently marked five plots of 0.5 × 0.5 m with a minimum between‐plot distance of 1 m to minimise the probability that bilberry ramets in neighbouring plots had clonal subterranean connections (Albert et al., 2003) and to maximise within‐block environmental variation (Figure A1). We rejected plots that (i) included tree stems; or (ii) had a cover of more than 50% of logs or stones. At the highest‐situated block, few ramets produced flowers and berries. Here, we systematically rejected plots that lacked fertile ramets, until a minimum one plot with at least one fertile ramet had been included. Rejected plots were substituted by new plots according to a fixed priority list.

#### 2.3.1 Species composition and cover of bilberry

We divided each plot into 16 subplots, each 0.0156 m^2^. In June–July 2017, we recorded the presence or absence of vascular plants, bryophytes and lichens in each subplot and calculated subplot frequency as a measure of species' abundance. We also visually estimated percent cover of bilberry in each plot. In total, 27 vascular plant, 24 bryophyte and 12 lichen species were recorded in the 50 plots (the mean number of species per plot was 11).

#### 2.3.2 Bilberry vegetative growth

We tagged selected bilberry ramets in each plot by applying a split, coloured Hama maxi bead to the main twig of each ramet (Hegland et al., 2010). Ramet selection, performed in 2017, started in one of the four central subplots and continued in an anticlockwise manner by including additional subplots until eight ramets were included (Figure A1). The total number of ramet tagged in 2017 was 272, of which 15 could not be relocated at the 2018 census. That year we included all fertile ramets in each plot, thereby increasing the total number of tagged ramets by 108. A total of 46 and 40 plots contained berry‐producing ramets in 2017 and 2018, respectively. In 2017 and 2018, we measured plant height (H), stem diameter (DS) and number of annual shoots (AS) for all tagged ramets and calculated dry mass (DM) of each ramet as a nondestructive estimate of plant size, using the formula described by Hegland et al. (2010): log_2_ (DM) = 1.41700 × log_2_ (DS) + 0.97104 × log_2_ (H) + 0.44153 × log_2_ (AS +1) –7.52070. The formula is based upon a model developed from a representative, destructively sampled data set (*R* ^2^ = 0.944, *n* = 150).

#### 2.3.3 Bilberry reproduction

Each year from 2017 to 2020, we counted the total number of berries produced in each 0.25 m^2^ plot and multiplied this number by four to express berry production as berries per square metre (m^−2^). In 2017 and 2018, we also counted seeds. We collected all berries produced by the tagged ramets in each plot, dried them in a drying cabinet at 70°C for 36 h and weighed them individually. For each plot, the berry closest to plotwise mean mass was selected, dissected under a stereo microscope and the number of mature (>1 mm long, filled) and nonmature seeds (aborted seeds or unfertilised ovules; <1 mm, flat or negligibly swollen) were counted. The counts were used to calculate two measures of seed production per plot: the number of mature seeds, and the fraction of mature to total (mature and immature) seeds, per berry (Pato & Obeso, 2012a).

#### 2.3.4 Environmental variables

A set of 16 environmental variables that could potentially influence species composition and bilberry performance, was recorded for each of the 50 plots based on field measurements or soil samples taken in 2017 (Table A.1). We recorded soil chemical and physical variables for the 50 plots from soil samples obtained by mixing four soil subsamples taken from the upper 10 cm of the humus layer in each plot. Soil samples were dried at 35°C for 10 days in drying cabinets before sifting (2 mm mesh width). pH and soil organic matter (SOM) were measured at the soil laboratory at Western Norway University of Applied Sciences, Sogndal, by procedures described by Krogstad (1992). All other soil analyses were carried out at the soil laboratory of the Norwegian University of Life Sciences, Ås. Total N was determined by the Dumas method. Other soil elements were extracted in a NH_4_NO_3_ solution (Stuanes et al., 1984) for the determination of exchangeable element concentrations by ICP. Among these, we used elements regarded as macronutrients, micronutrients or toxic to plants (P, K, Al, Ca, Fe, K, Mg, Mn, S and Zn) for further analyses. We recalculated all elements as ppm (parts per million) by multiplication with 1/SOM (Økland, 1988). We recorded soil moisture in August 2017 after 4 days without rainfall, using an AT Delta‐T moisture metre, type HH2 SM300 v 4.0. The mean moisture, used in analyses, was calculated from four measurements per plot. Soil depth was measured as the distance a steel rod (diameter 1 cm) could be driven into the soil at eight fixed positions 10 cm outside the plot border. The median of these eight values was used in the analyses. The Heat Index was calculated according to Heikkinen (1991) by measuring aspect and slope (0°–360° scale) using a clinometer compass held at a position considered representative for each plot. We measured canopy openness by use of a convex, spherical densiometer with 24 squares (Lemmon, 1956). Canopy openness (Light) was estimated as the number of squares not containing canopy.

#### 2.3.5 Climatic variables

We included seven climatic variables in the variation partitioning analyses. By comprising both *Elevation* and *Squared elevation*, we allowed potential linear, as well as unimodal, relationships between elevation and bilberry performance. The latter five variables were included because they have previously been shown to relate to bilberry performance in various ways (Bokhorst et al., 2011; Hertel et al., 2018; Rixen et al., 2010; Selås et al., 2015), see Appendix S.1 and Tables A.2 and A.3. *Minimum January temperature* (JanMinT) was included since warm winters have been shown to reduce vegetative growth and affect berry production, and *Minimum temperature in the flowering period April–June* (MinTFlow), was included since cold periods in the flowering season have been shown to reduce berry production. *Growing days* (number of days with middle temperature >5°C; GDays) and *Growing days degrees* (daily mean temperature, summarised over growing days in May–August; GDDegr) were included because long and warm growing seasons have been shown to increase berry production and vegetative growth. Finally, *Precipitation in June–August* (Precip) was included since both wet and dry summers have been shown to reduce berry production.

We obtained data for temperature and precipitation from Sogndal airport (temperature only; station located 7 km S of the study site, at 497 m a.s.l.) and Selseng (precipitation only, 17 km NW of the study site, at 421 m a.s.l.). However, normal values (1960–1990) for these stations differed much from interpolated values for the nearby Sogndalsfjøra settlement (1 km W of the study site, 10 m a.s.l., Table A.2). We used these differences in normal values to estimate temperature and precipitation for Sogndalsfjøra in the study period (Appendix S.1) and then further extrapolated these estimates to the elevations of each of the 10 blocks, using the theoretical lapse rates for temperature: mean value per 100 m elevation for winter months (December–February): −0.56°C, and for summer months (June–August): −0.54°C (Laaksonen, 1976) and summer precipitation: +1.6 mm per 100 m elevation (Førland, 1979).

The study years differed with respect to climatic variables (Tables A.2 and A.3): 2018 had the longest, warmest and driest growing season, whereas growing seasons were shorter and colder in 2017 and 2020. In 2019, the growing season was exceptionally wet, with low temperatures during bilberry flowering. The estimated differences in the five climatic variables between the lowest and highest situated blocks (assumed to be equal among years) amounted to, respectively, 4.0°C for JanMinT, 3.8°C for MinTFlow and 11 mm for Precip. For the latter two variables (GDays and GDDegr), we included between‐years variation as described in Appendix S.1 and calculated the average difference and SD to be, respectively, 8 ± 3 days for GDays, and 452 ± 9 day‐degrees for GDDegr (Table A.3).

### 2.4 Statistical analyses

All statistical analyses were performed in R version 3.6.2 (R Development Core Team, 2019). We used four‐dimensional GNMDS (Global Non‐Metric Multidimensional Scaling; Minchin, 1987) as implemented in the vegan package of R, version 2.4‐2 (Oksanen et al., 2017) and split‐plot GLM analysis (Auestad et al., 2008) to extract the main gradient structure of the species composition in the 50 plots and to interpret the main gradients' relationship to elevation and environmental variables (Appendix S.2).

Mixed effects models were used to investigate the relation between elevation and three sets of bilberry performance variables: (i) vegetative, two variables: estimated mean ramet size (DM; g) in 2017 and 2018, and bilberry cover (m^2^) in 2017; (ii) berry production (berries per m^2^) over the 4 years 2017–2020 and (iii) seed production (two estimates per berry: number, and fraction (%) of mature seeds), in 2017 and 2018.

We modelled bilberry performance variables (cover, ramet size, berry production, number and fraction of mature seeds) as functions of elevation. Bilberry cover (expressed as percentage, e.g. strictly bounded but nonbinomial) was logit‐transformed (Warton & Hui, 2011), and ramet size was log‐transformed, prior to modelling by the lmer function with identity‐link and Gaussian errors. We used the glmer function with log link and Poisson errors to model berry production, and glmer with logit link and binomial errors to model number of seeds and fraction of mature seeds. We performed the lmer and glmer analyses and parameterised the models using the R package lme4 (Bates et al., 2015). We accounted for the spatially nested sampling design by specifying ramet nested within plot within block as random effect in models for ramet size, and plot nested within block in models for cover, berry production and number and fraction of mature seeds. Eventual overdispersion was handled by including a unique code for each plot in the random variables (Elston et al., 2001). Singularity problems allowed us to include only the overdispersion variable for number of mature seeds, and plot number and the overdispersion variable for proportion of mature seeds. We modelled a polynomial functional relationship to account for potential unimodal relationships between elevation and the various response variables. In line with the principle of parsimony, we simplified the models into the minimal adequate model, using a backward elimination procedure with likelihood ratio tests (Crawley, 2013).

**FIGURE 2:**
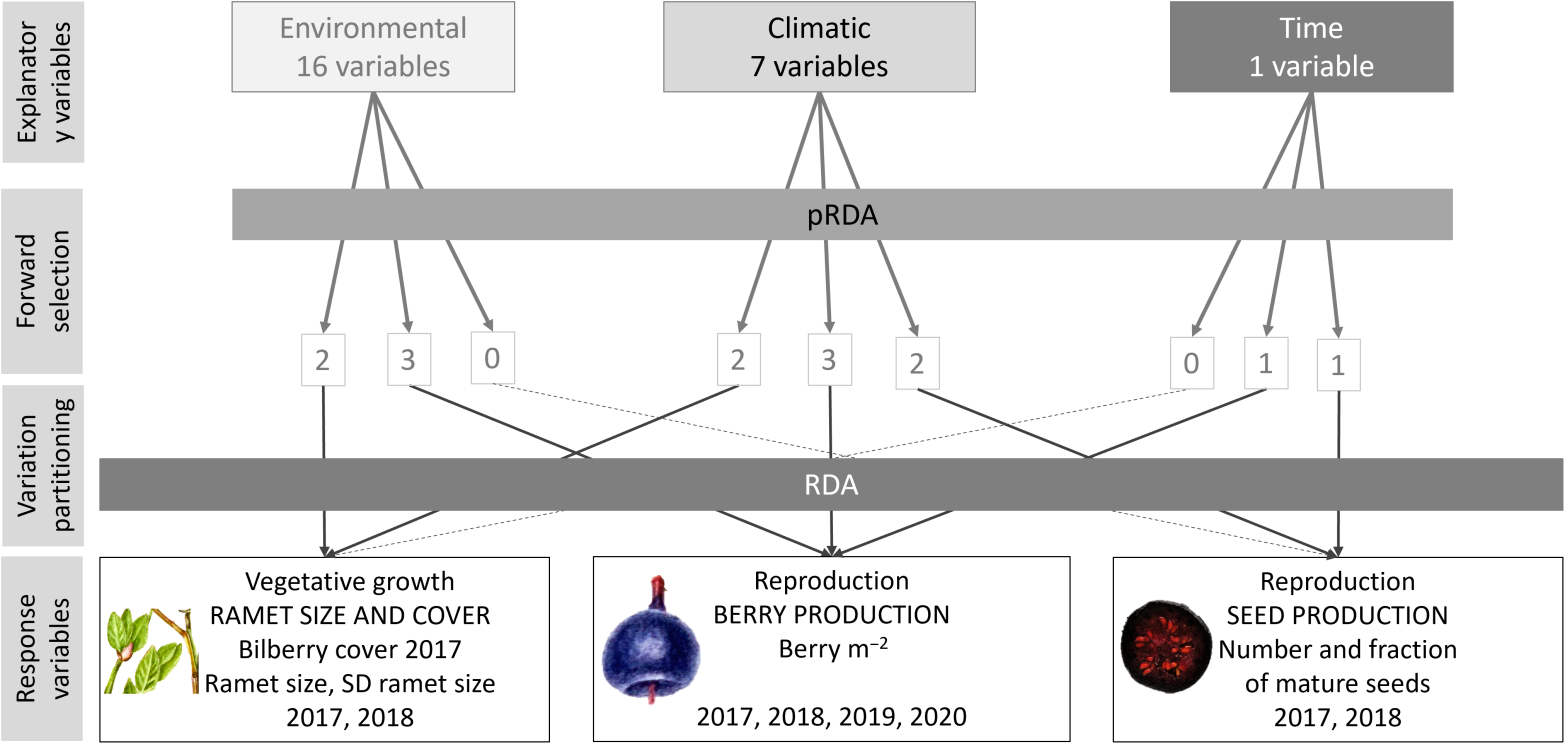
Conceptual diagram showing the four steps of the variation partitioning procedure that we used to quantify the unique and shared contribution of three groups of explanatory variables (environmental and climatic variation, and time; top) to explaining variation in the three sets of bilberry performance response variables (one vegetative; ramet size and cover, and two reproductive; berry production and seed production; bottom). We used partial constrained ordination (pRDA, Table A.5, see text for details) to identify the significantly contributing explanatory variables for each response variable set. The number of identified variables per set of performance variables is given in the nine white boxes.

Variation partitioning (Borcard et al., 1992; Økland, 1999) based on repeated partial constrained ordinations was used to quantify the relative importance of three groups of explanatory variables on bilberry performance (Figure 2): environmental variation (E; 16 variables), climatic variation (C; seven variables) and time (T; one categorical variable, with two or four levels, depending on the data for the response variable, included to reflect temporal climatic variation). All variables were zero‐skewness transformed before analyses (Økland, 2003). As response variables in the variation partitioning, we used the same bilberry performance variables sets as in the mixed effect models: (i) vegetative response, (ii) berry production and (iii) seed production (note that set (i) was expanded with the variation (SD) of bilberry cover), cf. Figure 2. We matched the different response variable sets with the appropriate subset of climatic data: for berry production, we included data for all years (2017–2020), whereas for vegetative growth and seed production, we included data for 2017 and 2018.

For each set of response variables and for each explanatory variable group, we used the forward selection procedures in redundance analysis (RDA; ter Braak, 1987), as implemented in vegan (Oksanen et al., 2017) to find, separately for each group of explanatory variables (Figure 2, upper row), the variables that independently contributed significantly (*p* < .01) to the variation in each set of response variables (Table 1). Two environmental and two climatic variables were selected for vegetative performance; three environmental and three climatic variables, and time, were selected for berry production; and two climatic variables and time were selected for seed production (numbers in boxes in Figure 2). Groups for which no significant variable was found for a given set of response variables were left out from the variation partitioning analysis of the data set in question.

**TABLE 1:** Results from the forward selection procedure in pRDA to exclude redundant variables in each of the three sets of explanatory variables: climatic (seven candidates), environmental (16 candidates), and time (one candidate).

	Species composition 2017			Vegetative performance 2017–2018			Seed production 2017–2018			Berry production 2017–2020		
	TI = 2.656			TI = 0.171			TI = 0.195			TI = 0.071		
	inertia	*F*	*p*	inertia	*F*	*p*	inertia	*F*	*p*	inertia	*F*	*p*
Climatic variation												
Elevation	0.098	2.06	.0078			ns			ns	0.017	63.92	>.0001
Elevation^2			ns			ns			ns			ns
Min T Jan			ns	0.015	9.42	.0003			ns			ns
Min T Flow			ns			ns			ns			ns
Growth days (GD)			ns			ns			ns			ns
Growth days degrees (GDDegr)	0.322	6.63	>.0001			ns			ns			ns
Precipitation May‐August (Prec)			ns	0.014	9.34	.0005	0.115	79.85	>.0001	0.01	44.05	>.0001
Local environmental												
Heat index (HI)	0.129	2.73	.0005			ns			ns	0.003	11.04	>.0001
Soil depth (median)			ns			ns			ns			ns
Light (mean)			ns	0.019	11.9	>.0001			ns			ns
Soil moisture (mean)	0.194	3.79	>.0001			ns			ns	0.01	33.62	>.0001
Soil organic C			ns			ns			ns			ns
pH	0.161	3.29	.0002			ns			ns			ns
totalN			ns	0.015	10.4	.0004			ns	0.005	16.7	>.0001
Al			ns			ns			ns			ns
Ca			ns			ns			ns			ns
Fe			ns			ns			ns			ns
K			ns			ns			ns			ns
Mg			ns			ns			ns			ns
Mn			ns			ns			ns			ns
P			ns			ns			ns			ns
S			ns			ns			ns			ns
Zn			ns			ns			ns			ns
Time												
Year	Not included					ns	0.101	105.8	>.0001	0.012	13.77	>.0001

*Note*: TI denotes the total inertia of the analysis. Inertia, *F*‐values and *p*‐values are given only for variables that gave significant and independent contribution (*p* < .01) to the variation in each of the three response data sets.

Abbreviations: Gdays, Growing days; GDDegr, Growing days degrees; Prec, Precipitation May–August.

For each of the three sets of response variables, we performed a sequence of (partial) RDAs to quantify the unique and shared contributions of each explanatory variable group to explain the variation in the response. Variation components are reported as fractions of the total explained variation, as recommended by Økland (1999). Moreover, we interpreted the ratio of the total variation explained (TVE) and the total inertia (TI) as a measure of the minimum, rather than the total explained variation in the response variable, since the remaining, ‘unexplained’ variation (1 − TVE/TI) may include up to 50% of variation that is due to lack of fit of data to the underlying, essentially linear, statistical model (Økland, 1999).

## 3 RESULTS

### 3.1 Species composition and environmental variation along the elevation gradient

#### 3.1.1 Gradient extraction and interpretation

**FIGURE 3:**
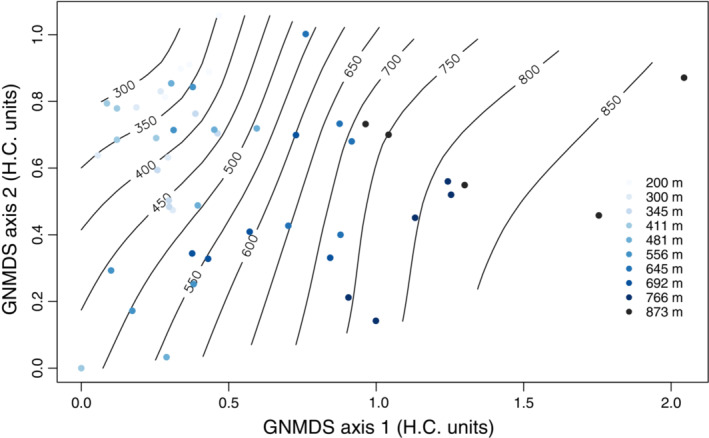
GNMDS ordination of the species compositional data set of 50 plots in 10 blocks, axes 1 and 2, scaled in H.C. units. Colours indicate plots from each of the 10 blocks, contour lines are isolines for elevation.

The main gradient in species composition (GNMDS axis 1; Figure 3), as interpreted by split‐plot GLM (Table 2), ran along the elevation gradient, and the majority of variation was found at block level. Blocks located at lower elevations had more closed canopies, drier and warmer conditions, and vegetation dominated by bilberry *V. myrtillus*, *H. splendens* and *Avenella flexuosa*. Blocks located at higher elevations had higher irradiance and colder, moister conditions. The vegetation was dominated by *E. nigrum* and *Cladonia arbuscula* agg., but still with considerable amounts of bilberry. At plot level, pH increased slightly along the axis (Table 2). Along the elevation gradient, soil moisture and SOM increased, whereas the Heat Index, pH and a number of soil nutrient concentrations (in particular P, but also K, Fe, Ca) decreased (Table A.1). Species turnover per vertical m was twice as high in the upper 200 m of the elevation gradient (above 650 m a.s.l.) as in the lower 450 m, as revealed by the first GNMDS axis (Figure 3).

**TABLE 2:** Output of split‐plot GLM analyses of GNMDS axis 1.

Explanatory variable	Block				Plot			
	Sum of squares = 8.06				Sum of squares = 1.40			
	FVE = 0.85				FVE = 0.15			
	SS_expl_/SS_blokk_	*F*	*p*	coeff	SS_expl_/SS_plot_	*F*	*p*	coeff
Elevation (m)	**0.70**	**18.36**	**<.01**	**+**	ns			
Heat index	0.44	6.20	<.05	−	ns			
Soil depth (median)	ns				ns			
Light	0.49	7.55	<.05	+	ns			
Soil moisture (mean)	**0.59**	**11.47**	**<.01**	+	ns			
Soil organic C	ns				ns			
pH	ns				**0.16**	**7.49**	**<.01**	**+**
totalN	ns				ns			
Al	ns				ns			
Ca	ns				ns			
Fe	ns				ns			
K	ns				ns			
Mg	ns				ns			
Mn	ns				ns			
P	ns				ns			
S	ns				ns			
Zn	ns				ns			

*Note*: Relationships between the main vegetation gradient (GNMDS plot scores; response variable) and explanatory variables (elevation and 16 local, environmental variables), evaluated at two grain levels (block and plot) by split‐plot GLM (identity link, normal errors).

Total sum of squares (SS) and fraction of total variation explained (FVE) for block and plot level are given in the upper part and values for the individual variable in the lower part of the table. Block level had nine, and plot level 40 degrees of freedom. The variation explained (SS_expl_/SS_grain level_) by each explanatory variable is given for both block and plot level. *p* < .01 are given in bold. Model coefficient (coeff) show the sign of the relationship (+/−).

### 3.2 Bilberry growth: cover 2017 and ramet size 2017 and 2018

#### 3.2.1 Variation along the elevation gradient

**FIGURE 4:**
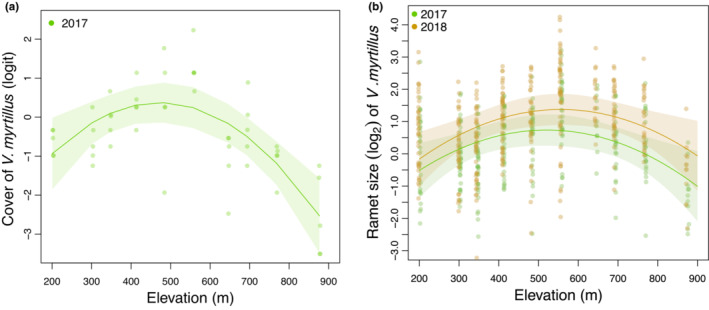
Variation in bilberry vegetative performance along the elevation gradient recorded in 2017 as bilberry cover (logit; %) (a), and in 2017 and 2018 as bilberry ramet size (log_2_; g) (b). Envelopes indicate 95% confidence intervals.

Bilberry cover showed a significant, unimodal pattern along the elevation gradient, peaking at intermediate elevations with a maximum cover of 90% at 550 m a.s.l. (Figure 4a; Table A.4). Also, bilberry ramet size was unimodally related to elevation, in both 2017 and 2018. Ramets were significantly larger in 2018 than in 2017 (Figure 4b; Table A.4).

#### 3.2.2 Variation partitioning of explanatory variables

**FIGURE 5:**
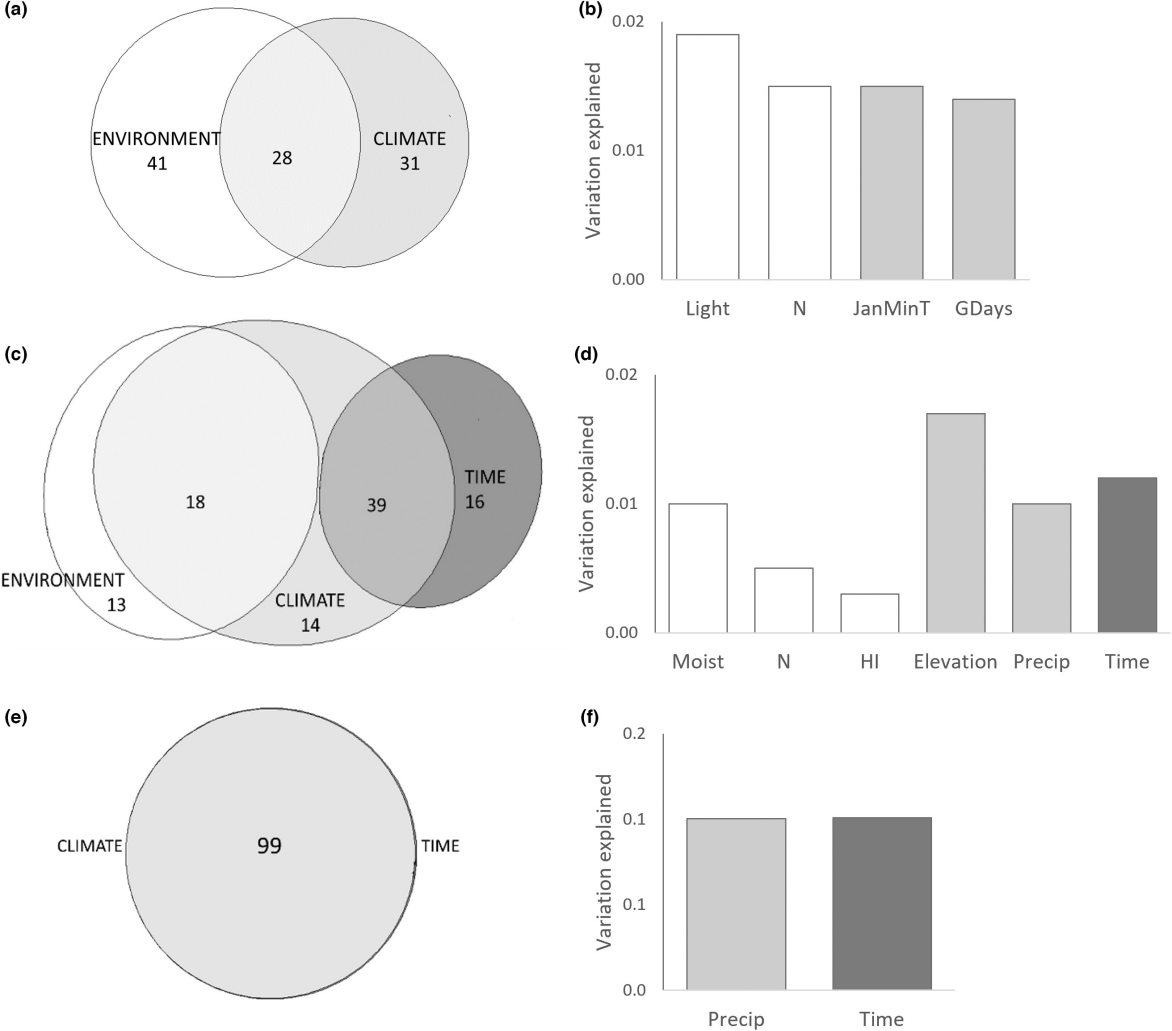
Results for partitioning of the variation in bilberry performance between three groups of variables: environment (white), climate (pale grey), and time (dark grey); for ramet size and cover (a, b), for berry production (c, d), and for seed production (e, f). Venn diagrams (a, c, e) show the relative contribution of environment, climate and time. Histograms (b, d, f) show the individual contribution of the independently, significantly (*p* < .01) contributing variables.

A minimum of one‐third of the total variation in vegetative performance (ramet size, variation in ramet size (SD) and cover of bilberry) was accounted for by the analysed variables (total inertia = 0.17, TVE = 0.05, Figure 5a; Tables A.4 and A.5). Environmental conditions, light and N totally explained 69% of TVE, 28% together with climate and 41% alone (Figure 5b). Climatic variation, represented by temperature‐related climatic variables JanMinT (winter cold) and GDays (length of growing season) explained a total of 59% of TVE and 31% alone (Figure 5b; Tables A.4 and A.5). Time did not explain variation in ramet size, in accordance with the similar patterns of variation in ramet size in the 2 years (Figure 4b).

### 3.3 Berry production 2017–2020

#### 3.3.1 Variation along the elevation gradient

**FIGURE 6:**
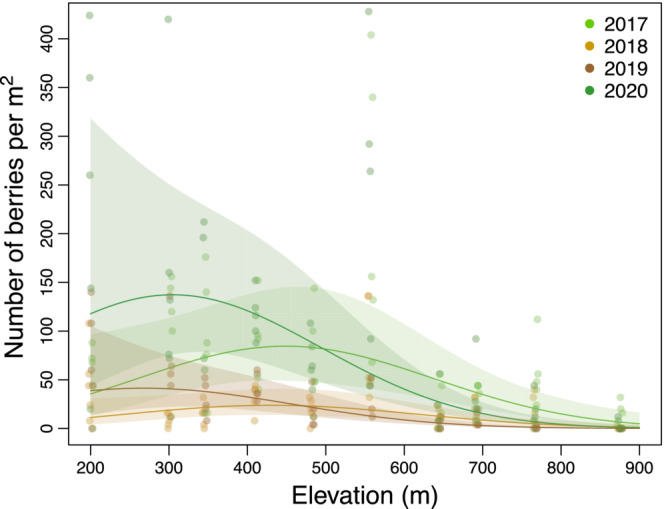
Berry production (number of berries per m^2^) along the elevation gradient 2017–2020. Envelopes indicate 95% confidence intervals.

Plotwise berry production (number of berries per m^2^) varied from 0 to 428, and showed a skewed unimodal relationship to elevation, peaking at elevations of 300 and 500 m a.s.l. in 2020 and 2017, respectively (Figure 6). However, in the dry year (2018) and the cold and wet year (2019), relationships were close to linear (Figure 6; Table A.5). In these years, total annual berry production in the 50 plots was much lower (1264 and 1348, respectively) than in years 2017 and 2020 (3848 and 5036, respectively).

#### 3.3.2 Variation partitioning of explanatory variables

More than half of the total variation in berry production was accounted for by the analysed variables (total inertia = 0.07, TVE = 0.04). Climatic variation (represented by elevation and precipitation) accounted for 77% of TVE, 39% shared with the environmental variables soil moisture, N and HI, and 18% shared with time (Figure 5c,d; Tables A.4 and A.5). Environmental variables accounted for 52% of TVE, of which 13% unshared, while time accounted for 34% of TVE, of which 16% unshared with other variable groups.

### 3.4 Seed production 2017 and 2018

#### 3.4.1 Variation along the elevation gradient

**FIGURE 7:**
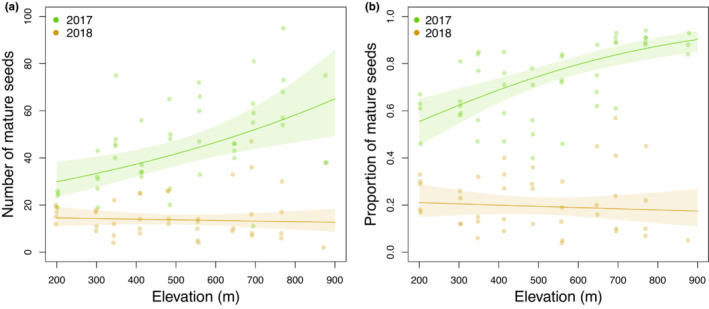
Seed production along the elevation gradient in 2017 and 2018, expressed as the numbers (a) and the fraction (b) of mature seeds per berry. Envelopes indicate 95% confidence intervals.

The number of mature seeds per berry varied from 2 to 92. We found significantly more fertile seeds, and a larger fraction of fertile seeds per berry, in 2017 than in 2018 (Figure 7a,b; Table A.5). This accorded with the patterns found for berry production in these 2 years (Figure 6). The number and fraction of fertile seeds per berry increased along the elevation gradient in the normal year 2017 but not in the dry year 2018.

#### 3.4.2 Variation partitioning of explanatory variables

A minimum of half the total variation in seed production was accounted for by the analysed variables (total inertia = 0.20, TVE = 0.10). No environmental variables explained a significant fraction of the variation, all explained variation was accounted for by climate variables (represented by precipitation; Figure 5e,f). This indicated an interaction effect between year and precipitation on seed production, in accordance with Figure 7b and Tables A.4 and A.5.

## 4 DISCUSSION

Although observational studies cannot reveal causative effects, our study design (50 plots in 10 sites, covering an elevation range of 675 m over a 2.7 km horizontal range that corresponds to an upwards temperature decline of ca. 3.8°C and an estimated increase of ca 11 mm in precipitation in June–August) provided a broad and relevant platform for exploring the performance of the boreal key species bilberry. Furthermore, there was considerable variation in species composition along the elevation gradient: only ca. 50% of the species from the lowermost plots also grew in the uppermost plots. Species compositional variation is a pivotal attribute of any ecosystem, as it summarises the outcomes of all important ecological processes (Clewell & Aronson, 2007; Sundqvist et al., 2013). The variation in species composition was particularly large close to the tree line, in the upper third of the elevation gradient. The decreasing tree cover towards the tree line coincided with larger variation in several environmental variables including light and exposure to wind, and potentially also soil nutrients (Helbach et al., 2022), all likely to contribute to increased variation in species composition on the forest floor (Barbier et al., 2008).

### 4.1 Environmental and climatic variation affects bilberry performance along the elevation gradient in the boreal forest

We find bilberry growth (cover and ramet size) to peak at ca 500 m, most likely due to an optimal combination of summer temperatures (decreasing with elevation) and precipitation levels (increasing with elevation) that gives rise to a beneficial climate at this level (Eldegard et al., 2019; Selås et al., 2015). Bilberry's clonal structure and perennial life history may dampen climate effects on growth (Gerdol et al., 2013). Nevertheless, whereas ramet size decreases towards both higher and lower elevations, cover drops more towards high (>700 m) than towards low (<300 m) elevations. We interpret this as an effect of the uphill lowering of temperatures, known to curb vegetative growth (Fernández‐Calvo & Obeso, 2004). However, the earlier snowmelt at lower elevations may also affect bilberry growth negatively because the lack of a stable snow cover in early spring breaks the deep winter dormancy, making the ramets more exposed to spring frost bud damage (Bokhorst et al., 2011).

Contrary to bilberry growth, bilberry berry production shows larger variation between years and along the elevation gradient, peaking at different elevations in different years. For bilberry, long and moderately warm growing seasons enhance berry production (Boulanger‐Lapointe et al., 2017; Selås, 2000; Selås et al., 2015), whereas particularly dry (or wet) growing seasons curtail berry development (Boulanger‐Lapointe et al., 2017; Selås, 2000; Selås et al., 2015). We observed peaks at middle (500 m) or low (300 m) elevations in ‘normal years’, but linear response curves in dry, or cold and wet seasons. Covariation between climatic conditions (weather cues) and berry production, both fluctuating among years, has led to proposals of bilberry as a masting species (Boulanger‐Lapointe et al., 2017; Miina et al., 2009; Selås, 2000). However, both the strict and the normal definition of masting species (sensu Kelly, 1994) require not only an ultimate (weather‐related) cue, but also a proximate cue, that is an evolutionary advantage of variable berry production, demonstrated through, for example resource switching (Herrera et al., 1998). No such pattern has yet been demonstrated for bilberry. Typical masting species are wind‐pollinated and wind‐dispersed trees (Herrera et al., 1998; Kelly, 1994), which show regional, rather than local or fine‐scale, patterns of variation in seed production. Our finding that the elevation at which berry production peaks varies among years, further contradicts the notion of bilberry as a masting species.

Bilberry has been shown to extend its distribution uphill in alpine areas, in line with many other boreal forest species (Klanderud & Birks, 2003; MacDougall et al., 2021; Steinbauer et al., 2018). However, at the same time, no negative trend is reported from lowland boreal forests, thus suggesting that the species persists, and widens its elevational niche (MacDougall et al., 2021). The explanation for this may be twofold: the species' slow clonal growth may prevent a rapid extinction from the lowlands when the climate gets warmer, while at the same time causing a build‐up of an extinction debt (Bertrand et al., 2011; Vellend et al., 2006).

Bilberry's alpine expansion, on the contrary, may be due to influx of berries and seeds from the boreal zone. Despite large seed production, bilberry seedlings are surprisingly rarely observed at the forest floor (García‐Rodríguez & Selva, 2021; Welch et al., 2000). This may be due to limited amounts of microsites with bare soil, bilberry's preferred germination substrate. Such microsites become increasingly abundant above the tree line. However, bilberry's demand for relatively high soil temperatures to germinate (>20°; Baskin et al., 2000), combined with slow germination (average of 61 days; Steyaert et al., 2019) should be expected to effectively obstruct germination of locally produced seeds in alpine areas. This opens for the possibility that boreal bilberry populations support alpine bilberry populations through regional source‐sink population dynamics (Eriksson, 1996): mature bilberry berries and seeds produced in boreal forest populations in July may effectively be transported upwards by berry‐eating birds and mammals (García‐Rodríguez et al., 2022) to become deposited in the alpine zone, in moist and nutrient‐rich dung, perfect for germination. This happens at a time when seeds produced by alpine populations are still immature. Gut passage has moreover been shown to reduce germination time to an average of 20 days (Steyaert et al., 2019), allowing the deposited bilberry seeds to germinate within the short alpine growing season.

Bilberry's alpine expansion and its boreal forest persistence can be interpreted as examples of vegetation disequilibrium (Svenning & Sandel, 2013) related to the established extinction debts. In years to come, a slow repayment of such debts must be expected and considered when assessing the relationships between climate and performance of a range of key species. This would imply that species such as bilberry eventually would disappear from lower elevations as a delayed response to ongoing climate warming.

The variation partitioning analyses indicate that not only climatic, but also environmental variation affects bilberry performance, uniquely and in concert. Environmental variables relating to N and light are identified as particularly important for ramet size and cover, in accordance with previous findings (Gerdol, 2005; Manninen et al., 2009; Nielsen et al., 2007). Similarly, variation in berry production is explained by climate as well as by the environmental variables soil moisture, N and heat. Soil moisture varies in space, as well as between years, as a response to inter‐annual, climatic variation. The influence of this fine‐scaled spatial mosaic of variation in soil moisture on berry production has, however, hardly been studied. Økland (1996) suggest that soil moisture is a direct driver of variation in berry production in boreal forest landscapes. Ramets in moist microsites (depressions) and north‐facing slopes optimise berry production in dry years, whereas ramets on shallow soils and in south‐facing sides produce more berries in wet years (personal observation, I. Auestad and K. Rydgren). Berry production hence peaks in different parts of the boreal forest landscape in different years (Kilpeläinen et al., 2016). We support the call of Bogdziewicz et al. (2020) for studies that reveal the true nature of key species' reproductive patterns, to understand the difference between true masting behaviour, and variation in fruit and seed production for reasons such as environmental variation.

Seed production per berry increases upwards in a normal, but not in a dry summer, and variation in precipitation among years alone explains this variation in our analysis. We found no relationship between seed production and the measured environmental variables. Few studies have investigated this relationship, but Eckerter et al. (2019) found a significant increase in both the number and fraction of mature seeds from shaded (5%–15% light) to experimentally opened (26%–40% light) plots, and attributed this to differences in pollinator activity. Cold and wet climates are known to curb pollination activity (Fernández‐Calvo & Obeso, 2004). This may explain the results we got for the drought‐ridden summer of 2018, but our 2‐year record of seed production is too limited to allow further discussions of such patterns along the elevation gradient. Pollination is known to affect bilberry performance, vary along the elevation gradient (Sundqvist et al., 2013), and become less important towards higher altitudes (Hulshof et al., 2013). One reason for this is that pollinator numbers usually decrease with increased elevation in the alpine zone (Totland, 2001). However, in steep forests in the boreal zone, this pattern may be reversed. When bilberry growing at low elevation (200 m) flowers in April–May, pollinator numbers are much lower than a few weeks later, when bilberry growing at higher elevation (>500 m) starts to flower (S. J. Hegland and M. Gillespie, unpublished data). Accordingly, the rate of self‐pollination, known to curtail seed set in bilberry, may be higher in the dense clones at lower elevations than in sites with lower cover (Nuortila et al., 2002), which in our study area are found at higher elevations.

### 4.2 Variation partitioning—useful for climatic space‐for‐time‐substitutions along elevation gradients?

Our results show that not only climatic, but also environmental variables explain considerable amounts of variation in most aspects of bilberry performance, and that interpretation of climatic space‐for‐time studies performed along elevation gradients should be made with considerable care: variation along the elevation gradient tends to be explained by a wider range of variables than climatic ones.

Much of the variation in performance is explained by climate and environment in concert, a result reflecting that these two groups of external conditions interact in multiple ways. Lower temperatures at high altitudes curb plant nutrient supply rates from the soil by lowering nutrient mineralisation while at the same time SOM content is enhanced by decreased decomposition rates (Sundqvist et al., 2013). Moreover, there is a gradual, upward increase in soil moisture, and a decrease in canopy cover (Zellweger et al., 2020). The fraction of variation explained by environmental variables alone, not shared with climatic variables, may provide a rough indication of the appropriateness of the climatic space‐for‐time substitution approach along an elevation gradient; failing when variation due to environmental conditions, not shared with climate, explains a large fraction of the variation (in our case up to 41% of TVE). This must be expected to be the case in the boreal forests, where fine‐scale variation in, for example nutrients such as N, topography, shade and soil moisture (Økland, 1996) affects the performance of species in independent ways along an elevation gradient. In the case of the long‐lived dwarf‐shrub bilberry, space‐for‐time substitutions should be interpreted with care when investigating vegetative growth as well as berry production, since only seed production seems unrelated to variation in environmental conditions.

The variation partitioning approach allows estimation of the relationship between total variation explained and total inertia (TVE/TI; in our case 26%–59%), which is far below the levels reported by, for example Volis et al. (2011) in their study of variables impacting the translocation success for populations of *Iris atrofusca* (74.3%). However, as Økland (1999) points out: TVE/TI is a much used, but inappropriate measure of the variation explained in such analyses since ordination techniques (in our case RDA) contribute unknown amounts of ‘variation’ to total inertia. Økland (1999) shows that even with simulated data sets in which species replace each other regularly along one or two gradients, almost half of the unexplained variation represents lack‐of‐fit to the model, rather than ecologically meaningful variation. Hence, focus should be on the explained, rather than the unexplained variation in variation partitioning analyses and on the relative explanatory power (TVE) rather than on between‐study differences in the total variation explained (TVE/TI).

A series of methodological considerations should be made when elevational gradients are used for climatic space‐for‐time studies. First, the position of the tree line along the studied elevation gradient should be highlighted, to enhance transferability of results among studies carried out in, for example the Alps (tree line ca. 1500–2200 m a.s.l.) and in Fennoscandia (tree line varying from ca 1200 m a.s.l. in central southern parts and decreasing to sea level in the north). Next, a sufficient number of sites should be included along the gradient, to avoid confounding variation among habitat types (differing in many aspects) with variation directly attributable to changes in climate. Our study design (10 sites distributed evenly along a 675 m elevation range) allowed us to explore the gradual change from dense, lowland forest up to the alpine tree line, rather than contrasting a few, different ecosystems along the gradient (e.g. Olsen et al., 2022; Pato & Obeso, 2012b). This enabled us to confirm both unimodal and linear trends in bilberry performance along the elevational gradient.

## 5 CONCLUSIONS

Our study shows that the variation in bilberry performance along an elevation gradient is related to local environmental as well as climatic variation, uniquely and in concert. This underlines the need for including both types of variation in studies of key species, particularly of clonal and long‐lived dwarf shrubs such as bilberry. Such studies, often performed using climatic space‐for‐time substitutions, may benefit from using variation partitioning to reveal the suitability of such substitution: if little variation is explained by climatic variables, or if most of the explained variation is related to local environmental variation alone, then climatic space‐for‐time substitutions should not be used. Hence, we recommend including both environmental and climatic variables in preliminary studies of variation along elevation gradients, and to supplement analyses of variation in plant performance by variation partitioning. A better understanding of how ongoing climate warming affects the performance of bilberry and other key species is pivotal for future successful management of boreal forests.

## AUTHOR CONTRIBUTIONS

**Inger Auestad:** Conceptualization (equal); formal analysis (equal); investigation (equal); visualization (equal); writing – original draft (lead); writing – review and editing (lead). **Knut Rydgren:** Conceptualization (equal); formal analysis (lead); investigation (equal); methodology (equal); visualization (equal); writing – original draft (supporting); writing – review and editing (equal). **Rune Halvorsen:** Formal analysis (equal); writing – review and editing (equal). **Ingrid Avdem:** Investigation (equal); writing – review and editing (supporting). **Rannveig Berge:** Investigation (equal); writing – review and editing (supporting). **Ina Bollingberg:** Investigation (equal); writing – review and editing (supporting). **Oline Lima:** Investigation (equal); writing – review and editing (supporting).

## Supporting information


Appendix S1

## Data Availability

The data that support the findings of this study are openly available in Dryad at https://datadryad.org/stash, reference number doi: 10.5061/dryad.j9kd51cj2.
