# Supplementary material for: Use climatic space‐for‐time substitutions with care: Not only climate, but also local environment affect performance of the key forest species bilberry along elevation gradient
Source: Ecol Evol. 2023 Aug 16;13(8):e10401. doi: 10.1002/ece3.10401 (PMC10432774; doi:10.1002/ece3.10401)
Supplement: Supplementary file 1 — Appendix S1 [file ECE3-13-e10401-s001.docx]

# Appendices for: Use climatic space-for-time substitutions with care: not only climate, but also local environment affect performance of the key forest species bilberry along elevation gradient

Inger Auestad, Knut Rydgren, Rune Halvorsen, Ingrid Avdem, Rannveig Berge, Ina Bollingberg and Oline Lima


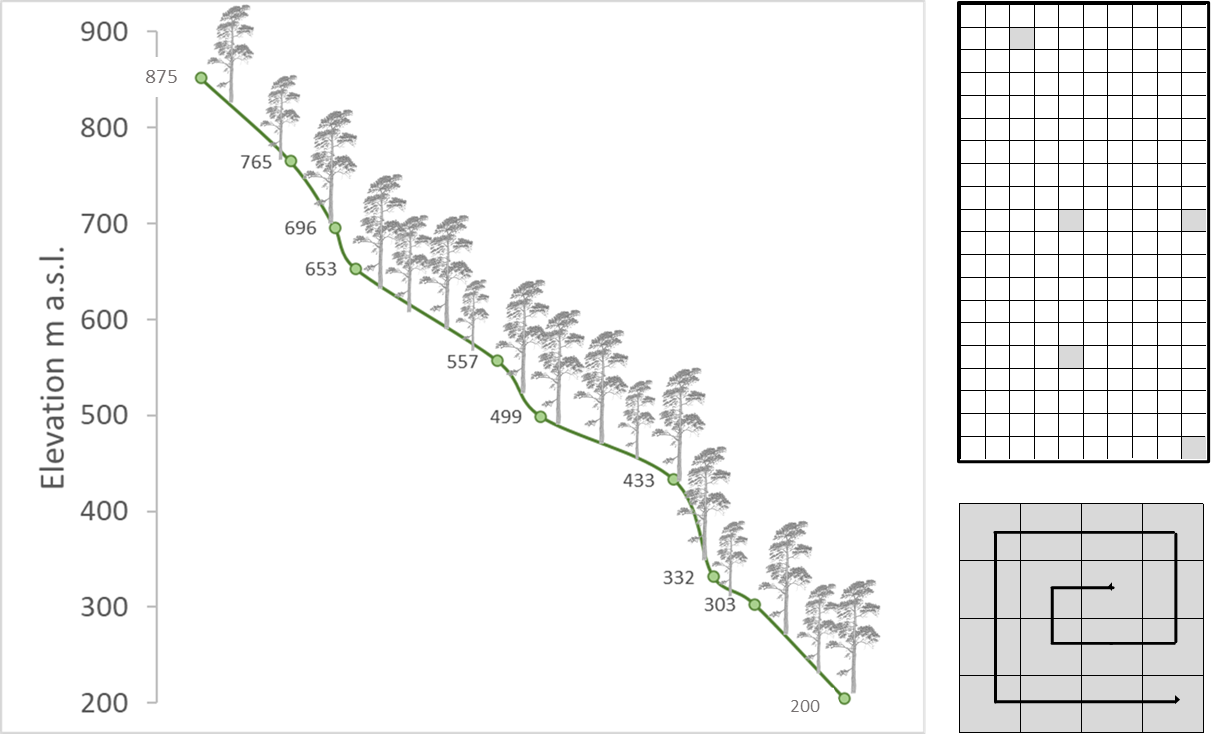


Figure A.1. The study design, indicating the elevation gradient from 200 to 875 m a.s.l. (a), the 5 x 10 m block placed at each elevation, with five 0.5 x 0.5 m plots (in gray) randomly placed within a single block (b), and one single plot with 16 subplots (c). The arrow in figure c demonstrates the procedure of selecting ramets, proceeding from the middle and anticlockwise.

Table A.1. Correlation matrix for elevation and the sixteen environmental variables. Kendall’s tau is given in the lower left half of the diagram, the corresponding P values in the upper right. Tau values > 0 are in brown, tau < 0 in blue, values >|0.25| are in bold. P values of 0.000 indicates P > 0.001.

|  | **Elevation** | **1.** | **2.** | **3.** | **4.** | **5.** | **6.** | **7.** | **8.** | **9.** | **10.** | **11.** | **12.** | **13.** | **14.** | **15.** | **16.** |
| --- | --- | --- | --- | --- | --- | --- | --- | --- | --- | --- | --- | --- | --- | --- | --- | --- | --- |
| Elevation |  | 0.012 | 0.422 | 0.112 | 0.009 | 0.007 | 0.003 | 0.082 | 0.013 | 0.000 | 0.030 | 0.056 | 0.001 | 0.894 | 0.000 | 0.101 | 0.001 |
| 1. Soil organic matter | 0.24 |  | 0.099 | 0.000 | 0.000 | 0.000 | 0.000 | 0.000 | 0.000 | 0.000 | 0.000 | 0.000 | 0.000 | 0.225 | 0.116 | 0.006 | 0.417 |
| 2. N | -0.08 | -0.16 |  | 0.195 | 0.114 | 0.189 | 0.362 | 0.122 | 0.042 | 0.004 | 0.000 | 0.189 | 0.019 | 0.008 | 0.640 | 0.831 | 0.086 |
| 3. Al | -0.16 | **-0.87** | 0.13 |  | 0.000 | 0.000 | 0.000 | 0.000 | 0.000 | 0.000 | 0.000 | 0.000 | 0.000 | 0.245 | 0.308 | 0.003 | 0.389 |
| 4. Ca | **-0.25** | **-0.87** | 0.15 | **0.87** |  | 0.000 | 0.000 | 0.000 | 0.000 | 0.000 | 0.000 | 0.000 | 0.000 | 0.362 | 0.071 | 0.008 | 0.213 |
| 5. Fe | **-0.26** | **-0.92** | 0.13 | **0.84** | **0.89** |  | 0.000 | 0.000 | 0.000 | 0.000 | 0.000 | 0.000 | 0.000 | 0.273 | 0.082 | 0.006 | 0.345 |
| 6. K | **-0.29** | **-0.82** | 0.09 | **0.70** | **0.78** | **0.83** |  | 0.000 | 0.000 | 0.000 | 0.001 | 0.000 | 0.000 | 0.427 | 0.061 | 0.131 | 0.345 |
| 7. Mg | -0.17 | **-0.81** | 0.15 | **0.88** | **0.81** | **0.80** | **0.70** |  | 0.000 | 0.000 | 7.603 | 0.000 | 0.000 | 0.126 | 0.462 | 0.003 | 0.225 |
| 8. Mn | -0.24 | **-0.75** | 0.20 | **0.68** | **0.77** | **0.75** | **0.70** | **0.64** |  | 0.000 | 0.000 | 0.000 | 0.000 | 0.398 | 0.050 | 0.010 | 0.259 |
| 9. P | **-0.44** | **-0.65** | **0.28** | **0.54** | **0.62** | **0.63** | **0.67** | **0.51** | **0.65** |  | 0.000 | 0.000 | 0.000 | 0.148 | 0.004 | 0.123 | 0.086 |
| 10. S | -0.21 | **-0.42** | **0.61** | **0.36** | **0.37** | **0.38** | **0.31** | **0.39** | **0.37** | **0.46** |  | 0.000 | 0.000 | 0.023 | 0.056 | 0.249 | 0.139 |
| 11. Zn | -0.19 | **-0.75** | 0.13 | **0.65** | **0.72** | **0.74** | **0.75** | **0.66** | **0.76** | **0.58** | **0.35** |  | 0.000 | 0.417 | 0.108 | 0.011 | 0.740 |
| 12. pH | **-0.31** | **-0.53** | 0.23 | **0.48** | **0.53** | **0.52** | **0.56** | **0.46** | **0.58** | **0.65** | **0.39** | **0.51** |  | 0.136 | 0.027 | 0.024 | 0.061 |
| 13. Soil depth_median | -0.01 | 0.12 | **-0.26** | -0.11 | -0.09 | -0.11 | -0.08 | -0.15 | -0.08 | -0.14 | -0.22 | -0.08 | -0.15 |  | 0.116 | 0.037 | 0.014 |
| 14. Soil moisture_mean | **0.53** | 0.15 | -0.05 | -0.10 | -0.18 | -0.17 | -0.18 | -0.07 | -0.19 | **-0.28** | -0.19 | -0.16 | -0.22 | -0.15 |  | 0.033 | 0.116 |
| 15. Light_mean | 0.17 | **-0.29** | 0.02 | **0.31** | **0.27** | **0.28** | **0.27** | **0.30** | **0.27** | 0.16 | 0.12 | **0.26** | 0.23 | -0.22 | 0.22 |  | 0.490 |
| 16. Heat Index | **-0.31** | -0.08 | 0.17 | 0.08 | 0.12 | 0.09 | 0.09 | 0.12 | 0.11 | 0.17 | 0.14 | 0.09 | 0.18 | -0.24 | -0.15 | 0.07 |  |

### Appendix S.1. Calculation of climatic variation variables

### a. Selection of climatic variation variables

In addition to elevation and squared elevation, we included five climate variables, based on the findings of Hertel *et al.* (2018), Bokhorst *et al.* (2011) and Rixen *et al.* (2010) that identified the best climatic variation predictors for bilberry vegetative and reproductive performance:

- minimum January temperature (JanMinT; warm winters have been shown to reduce vegetative growth and affect berry production),
- minimum temperature in actual flowering period in April-June (MinTFlow cold periods in the flowering season have been shown to reduce berry production),
- number of growing days (>5°C) in May–August (GDays; long and warm growing seasons have been shown to increase berry production and vegetative growth),
- growing days degrees or daily mean temperature, summarised over growing days in May–August (GDDegr; long and warm growing seasons have been shown to increase berry production and vegetative growth),
- precipitation in June–August (Precip; both wet and dry summers have been shown to reduce berry production).

To calculate these variables for each of the years 2017 – 2020, we needed the following data: minimum daily temperature for January, April, May, and June, mean daily temperature in May–August and cumulative precipitation for June–August. We also needed to identify the annually explicit flowering periods for bilberry. The calculations are described in section b, below.

### b. Extrapolations of variables from Selseng and Sogndal airport to Sogndalsfjøra town

All climatic variation data (temperature and precipitation) were extracted from the service *seklima* at met.no (2020). We acquired normal data for 1960–1990, as well as data for the four study years, from Sogndal airport (temperature only) and from Selseng station (precipitation only). For the normal period 1960-90, Sogndal airport (7 km distance, 497 m elevation) reported annual middle temperature of 3.4°C, and Selseng, (17 km distance, 421 m elevation) annual precipitation of 1543 mm (met.no), but these values varied much from intrapolated, normal values for Sogndalsfjøra town (1 km distance from study site, 10 m elevation); 6.7°C and 1025 mm.

We used the differences between climate normals (Selseng and Sogndal airport versus Sogndalsfjøra) as a way to “translate” measured climate variation during the study period to Sogndalsfjøra. We then further extrapolated these data to the elevational gradient (200–875 m) using the theoretical lapse ratios along elevations (Laaksonen, 1976); Førland (1979); see 3. Recalculation of temperature variables to elevations 200–875 m.

We extracted normal data (1960–1990) from the two climate stations, and the interpolated, normal data (1960–1990) for Sogndalsfjøra. For temperature data, we obtained daily values. We then calculated the difference between station and interpolated normal values (Table A2). All temperatures were higher (+2.7–4) for Sogndalsfjøra, whereas precipitation was lower (-87 mm).

We subtracted the respective differences from the observations for each of the years 2017, 2018, 2019 and 2020 (Table A2). These observations included daily observations of minimum temperature in January, April, May and June, mean daily temperature for May–August and sum of precipitation in June–August. These values, corresponding to 10 m elevation, were then used to recalculate the values for the ten elevations (200–875 m), see section c, below.

### c. Recalculation of temperature variables to elevations 200 – 875 m

We used the values for the six observations of climatic variation in the four years (given in the four last columns of Table A.2), to recalculate and establish values for the block-wise elevations of our study area (200–875 m) for five selected climatic variation variables (Table A.3).

To establish site-corrected temperatures, we used the theoretical lapse ratios given by Laaksonen (1976); -0.56°C per 100 m elevation (for January, and average for April–June, the latter covering the flowering season) and -0.54°C per 100 m elevation (average for May–August, covering the growing season).

To establish site-corrected precipitation, we used the theoretical lapse ratio established for W Norway by Førland (1979): +9 mm increase in annual precipitation per 100 m elevation. Since ca 180 mm of the annual precipitation (1025 mm) fell in June–August in the normal period of 1960–1990 (corresponding to ca 18%), we calculated the lapse ratio for June–August as 9 mm * 18% = 1.6 mm per 100 m elevation.

From this, we determined the following five variables for each year and elevation:

- *Minimum January temperature* (Jan Min T; ᵒC) was identified as the lowest of the daily January temperature values.
- *Minimum temperature in the flowering period* (Min T Flow; ᵒC) was extracted for annually explicit flowering periods for bilberry (in line with the findings of Hertel *et al.*, 2018) in the three years, for the ten elevations. We used observations of bilberry flowering onset made in an ongoing (2017–) phenology study in nearby Kaupanger (10 km distance), at three forest clearing study sites at 100, 450 and 850 m elevation (Gillespie and Hegland, unpublished data). Using linear regression, we calculated the relation between elevation and flowering onset in each year between 2017 and 2020 (r^2^ = 0.95–0.99). We also linearly regressed the average flowering duration from elevation, (r^2^ = 0.97), based on 2017 data from the same data set, and used this to identify flowering termination for the different elevations in each year.
- Minimum temperature in the flowering period was then identified as the lowest of the daily temperatures in the delimited flowering period for each year and elevation.
- *Number of growing days* (GDays) was identified as the number of days with adjusted and recalculated daily mean temperatures in May–August > 5°C.
- *Growth days degrees* (GDDegr; ᵒC) was identified as the summed temperature for the growing days.
- *Summed precipitation for June–August* (Precip; mm) was directly adapted from the initially established values.

Table A.2. Normal climate data (minimum temperature in January, April, May, June, August, mean temperature in May – August, and cumulative precipitation June – August) for stations, interpolation of data to Sogndalsfjøra, and the difference between stations and Sogndalsfjøra. These differences were used to calculate values for the six climatic variation variables in the four years of the study (2017 – 2020). 1 km distance to study site, 10 m elevation). See text for further explanation.

|  | Normal 1960-90 (stations) | Normal  1960-90 (Sogndalsfjøra**) | *Difference; stations - Sogndalsfjøra* | 2017 | 2018 | 2019 | 2020 |  |
| --- | --- | --- | --- | --- | --- | --- | --- | --- |
| Min T Jan (°C) | -5.6 | -1.6 | 4 | -10.2 | -8.8 | -7.8 | -4.9 |  |
| Min T April* (°C) | -0.2 | 2.7 | 2.9 | -2.5 | -9 | -1.8 | -1.9 |  |
| Min T May* (°C) | 4.5 | 7.8 | 3.3 | 1.9 | 4.9 | -0.9 | 2.7 |  |
| Min T June* (°C) | 11.2 | 14.0 | 2.8 | 4.9 | 7 | -0.5 | 7.3 |  |
| Mean T May-August (°C) | 10.8 | 13.4 | 2.7 | 13.3 | 16.2 | 13.9 | 13.9 |  |
| Prec. June-August (mm) | 278 | 191 | -87 | 220 | 284 | 406 | 264 |  |

** We included only the days bilberry flowered in April-June. ***1 km distance to study site, 10 m elevation

Table A.3. The five temperature variables included in our models in the four study years for elevations 200–875 m. Values were compiled from weather stations, adjusted to 10 m elevation at the study site, and recalculated to the ten different elevations. See text for further explanation.

|  | **Elev (m)** | **Min T Jan (°C)** | | | | **Min T Flow (°C)** | | | | **Growth days** | | | | **Growth days degrees (°C)** | | | | **Sum Precipitation (mm)** | | | |
| --- | --- | --- | --- | --- | --- | --- | --- | --- | --- | --- | --- | --- | --- | --- | --- | --- | --- | --- | --- | --- | --- |
| **Block** |  | **2017** | **2018** | **2019** | **2020** | **2017** | **2018** | **2019** | **2020** | **2017** | **2018** | **2019** | **2020** | **2017** | **2018** | **2019** | **2020** | **2017** | **2018** | **2019** | **2020** |
| **1** | 200 | -11.3 | -9.9 | -8.9 | -6.0 | 1.2 | 3.7 | -2.1 | -1.8 | 121 | 123 | 115 | 111 | 1521 | 1883 | 1619 | 1551 | 220 | 284 | 406 | 264 |
| **2** | 300 | -11.9 | -10.5 | -9.5 | -6.6 | 3.2 | 3.2 | -2.6 | -1.0 | 121 | 123 | 110 | 109 | 1456 | 1816 | 1533 | 1481 | 222 | 285 | 408 | 266 |
| **3** | 345 | -12.1 | -10.7 | -9.7 | -6.8 | 2.9 | 2.9 | -2.9 | 1.3 | 120 | 123 | 108 | 107 | 1422 | 1786 | 1496 | 1445 | 222 | 286 | 409 | 267 |
| **4** | 411 | -12.5 | -11.1 | -10.1 | -7.2 | 2.5 | 2.5 | -2.4 | 1.6 | 119 | 122 | 108 | 107 | 1374 | 1738 | 1458 | 1407 | 223 | 287 | 410 | 268 |
| **5** | 481 | -12.9 | -11.5 | -10.5 | -7.6 | 2.1 | 2.1 | -2.7 | 1.2 | 118 | 121 | 108 | 105 | 1325 | 1687 | 1417 | 1357 | 224 | 288 | 411 | 269 |
| **6** | 556 | -13.3 | -11.9 | -10.9 | -8.0 | 1.7 | 1.7 | 0.8 | 1.3 | 118 | 121 | 108 | 104 | 1277 | 1638 | 1373 | 1310 | 226 | 289 | 412 | 270 |
| **7** | 645 | -13.8 | -12.4 | -11.4 | -8.5 | 1.2 | 2.8 | 0.3 | 4.9 | 117 | 120 | 106 | 104 | 1215 | 1575 | 1312 | 1260 | 227 | 291 | 413 | 271 |
| **8** | 692 | -14.1 | -12.7 | -11.7 | -8.8 | 0.9 | 2.5 | 0.0 | 4.6 | 117 | 120 | 104 | 104 | 1185 | 1544 | 1275 | 1233 | 228 | 292 | 414 | 272 |
| **9** | 766 | -14.5 | -13.1 | -12.1 | -9.2 | 0.5 | 4.4 | -0.4 | -0.6 | 116 | 120 | 104 | 101 | 1134 | 1496 | 1233 | 1177 | 229 | 293 | 415 | 273 |
| **10** | 873 | -15.1 | -13.7 | -12.7 | -9.8 | -0.2 | 3.7 | 0.1 | -1.3 | 116 | 118 | 104 | 99 | 1067 | 1418 | 1173 | 1109 | 231 | 295 | 417 | 275 |

### Appendix S.2. Detailed description of the split-plot GLM analyses of GNMDS axis 1

We interpreted the main vegetation gradient by using the GNMDS plot score as response variable and one explanatory variable in turn as predictor in a split-plot GLM (generalised linear models, McCullagh & Nelder 1989; Venables & Ripley 2002) analysis, specifying error components at two hierarchical levels (Crawley 2002). The four-dimensional GNMDS was chosen after comparison with DCA and two-and three-dimensional GNMDS ordinations.

Explanatory variables included elevation, and the 16 local, environmental variables. Thus, statistical inference was obtained by considering plots as nested within blocks (50 plots within 10 blocks). We calculated the overall fraction of total variation explained (FVE) at both grain levels as the level sum of squares (SS_level_) divided by the total sum of squares (SS_total_) for the axis. The variation explained for each variable on each grain level was computed as SS for the variable (SS_variable_) divided by the SS for both grain levels (SS_grain level_). For each model, the sign of the coefficient c (positive or negative) indicated the sign of significant relationships. As numerous tests were performed, we restricted interpretation to strongly significant relationships (p-values of 0.01 or lower) to avoid making Type I errors, as recommended by (Crawley, 2013). Split-plot GLM with identity link function and normal distribution of errors (family = Gaussian) was performed by R, version 2.4-2 (Oksanen *et al.*, 2017).

*Table A.4. Results of linear mixed modelling of the effect of the three sets of explanatory variables (climatic variation, environment, and time) on response variables (measures of bilberry performance).* Model parameters estimates are obtained from the minimal adequate LME og GLMER models, assuming a normal error structure and accounting for spatial and temporal pseudoreplication. The first line in each table denotes the response variable level that is included in the intercept. Values for Estimate, SE and z-value*for the explanatory variables are tests of the differences from this level. Significant variables (P <0.001) are given in bold.

| Response variable | Fixed effects | Estimate | SE | z-value* | P |
| --- | --- | --- | --- | --- | --- |
| Vegetative: Bilberry cover | *Bilberry cover* | *0.32* | *0.27* | *1.18* |  |
| 2017 | Elevation | -0.41 | 0.19 | -2.24 | 0.060 |
|  | Elevation^2^ | -0.77 | 0.20 | -3.87 | **0.006** |
| Vegetative: Bilberry ramet | *Ramet size in 2017* | *2.26* | *0.39* | *5.71* |  |
| size 2017 and 2018 | Year (2018) | 0.95 | 0.12 | 7.94 | **<0.001** |
|  | Elevation | 0.24 | 0.27 | 0.89 | 0.400 |
|  | Elevation*^2^* | -0.67 | 0.29 | -2.28 | 0.054 |
| Reproductive: Berry | *Berry production in 2017* | *4.35* | *0.29* | *15.06* |  |
| production (m^-2^) 2017–20 | Year (2018) | -1.31 | 0.14 | -9.61 | **<0.001** |
|  | Year (2019) | -1.57 | 0.14 | -10.95 | **<0.001** |
|  | Year (2020) | -1.12 | 0.13 | -0.95 | 0.343 |
|  | Elevation | -0.46 | 0.21 | -2.21 | **0.027** |
|  | Elevation*^2^* | -0.60 | 0.21 | -2.89 | **0.003** |
|  | Year 2018: Elevation | -0.11 | 0.14 | -0.73 | 0.464 |
|  | Year 2019: Elevation | -1.05 | 0.16 | -6.69 | **<0.001** |
|  | Year 2020: Elevation | -0.84 | 0.14 | -6.02 | **<0.001** |
| Reproductive: number of | *No. mature seeds in 2017* | *3.76* | *0.07* | *57.24* |  |
| fertile seeds 2017 and 2018 | Year (2018) | -1.15 | 0.10 | -11.03 | **<0.001** |
|  | Elevation | 0.23 | 0.07 | 3.30 | **<0.001** |
|  | Year 2018: Elevation | -0.27 | 0.11 | -2.44 | **0.015** |
| Reproductive: fraction | *% fertile seeds in 2017* | *1.16* | *0.11* | *10.51* |  |
| fertile seeds 2017 and 2018 | Year (2018) | -2.59 | 0.16 | -15.94 | **<0.001** |
|  | Elevation | 0.60 | 5.02 | 5.02 | **<0.001** |
|  | Year 2018: Elevation | -0.66 | 0.18 | -3.80 | \| <0.001 \| \| --- \| |

* t-value for bilberry cover and bilberry ramet size

Table A.5. The variation explained (in percentage of total variation) by the three groups of explanatory variables environment (E), climatic variation (C) and time (T) on the three sets of response variables: bilberry ramet size /cover, berry density, and seed production. Single letters E, C and T denote the variation explained by the group in isolation. Combination of two letters (E&C, E&T, and C&T) denote the variation explained by these two groups in combination and E&C&T denotes the variation explained by all three groups. Time was not included for the analysis of species composition. TVE/TI equals the total variation explained divided by total inertia.

| Response variable set |  | E | C | T | E&C | C&T | E&T | E&C&T | TVE/TI |
| --- | --- | --- | --- | --- | --- | --- | --- | --- | --- |
| Bilberry ramet size and cover | | 41 | 31 | 0 | 28 | 0 | 0 | 0 | 29 (0.05/0.17) |
| Bilberry berry density | | 13 | 14 | 16 | 39 | 18 | 0 | 0 | 57 (0.04/0.07) |
| Bilberry seed production | | 0 | 0 | 1 | 0 | 99 | 0 | 0 | 50 (0.10/0.20) |

References

Bokhorst, S., Bjerke, J.W., Street, L.E., Callaghan, T.V., Phoenix, G.K., 2011. Impacts of multiple extreme winter warming events on sub-Arctic heathland: phenology, reproduction, growth, and CO_2_ flux responses. Global Change Biol. 17, 2817-2830.

Førland, E.J., 1979. Nedbørens høydeavhengighet. Klima 2, 2-34.

Hertel, A.G., Bischof, R., Langval, O., Mysterud, A., Kindberg, J., Swenson, J.E., Zedrosser, A., 2018. Berry production drives bottom–up effects on body mass and reproductive success in an omnivore. Oikos 127, 197-207.

Laaksonen, K., 1976. The dependence of mean air temperatures upon latitude and altitude in Fennoscandia (1921-1950). Annls Acad. scient. fenn. Ser A 3 geol.-geogr. 119, 1-19.

met.no, 2020. eKlima. Climatedata from Norwegian Meteorological Institute. Norwegian Meteorological Institute, Available from <http://www.eklima.met.no>.

Oksanen, J., Blanchet, F.G., Friendly, M., Kindt, R., Legendre, P., Minchin, P.R., O'Hara, R.B., Simpson, G.L., Solymos, P., Stevens, M.H.H., Szoecs, E., Wagner, H., 2017. vegan: Community Ecology Package. R package version 2.4-2, Available online at: <http://CRAN.R-project.org/package=vegan> (accessed on 3 July 2019).

Rixen, C., Schwoerer, C., Wipf, S., 2010. Winter climate change at different temporal scales in *Vaccinium myrtillus*, an Arctic and alpine dwarf shrub. Polar Res. 29, 85-94.
